# Updated checklist of the vertebrate fauna of the Doñana Biosphere Reserve, Spain

**DOI:** 10.3897/zookeys.1269.166028

**Published:** 2026-02-17

**Authors:** Jacinto Román, Alberto M. Arias, José L. Arroyo, Giulia Bastianelli, Javier Calzada, Miguel Clavero, M. Dolores Cobo, Carmen Díaz-Paniagua, Carlos Ibañez, Javier Juste, Antonio Martínez, Jesús Nogueras, Leónidas de los Reyes, Eloy Revilla, Rubén Rodríguez, Eduardo José Rodríguez-Rodríguez, José L. del Valle, Guyonne F. E. Janss, Zulima Tablado, Marcello D’Amico, Laetitia M. Navarro

**Affiliations:** 1 Estación Biológica de Doñana-CSIC, Américo Vespucio n° 26, 41092 Sevilla, Spain Estación Biológica de Doñana-CSIC Sevilla Spain; 2 Instituto de Ciencias Marinas de Andalucía-CSIC, Campus Rio San Pedro S/N, CP 11519, Puerto Real – Cádiz, Spain Instituto de Salud Carlos III Majadahonda Spain; 3 Departamento de Ciencias Integradas y Centro de Estudios Avanzados en Física, Matemáticas y Computación, Facultad de Ciencias Experimentales, Universidad de Huelva, 21071 Huelva, Spain Universidad de Huelva Huelva Spain; 4 Espacio Natural de Doñana, Centro Advo. El Acebuche s/n, 21760 Matalascañas, Huelva Instituto de Ciencias Marinas de Andalucía-CSIC Puerto Real – Cádiz Spain; 5 Epidemiología y Salud Pública, CIBERESP, Instituto de Salud Carlos III, Ctra. Majadahonda-Pozuelo Km 2, 28220 Majadahonda, Madrid, Spain Espacio Natural de Doñana Matalascañas Spain; 6 TRAGSATEC-Doñana, C/ Montehigo 1, 21730 Almonte, Huelva, Spain TRAGSATEC-Doñana Almonte Spain; 7 Sociedad Ibérica para el Estudio y Conservación de los Mamíferos, Hacienda Miraflores, Parque Miraflores s/n, 41015 Sevilla, Spain Sociedad Ibérica para el Estudio y Conservación de los Mamíferos Sevilla Spain

**Keywords:** Biodiversity, Man and the Biosphere Programme, species richness, species diversity, taxonomic diversity, UNESCO Biosphere Reserve

## Abstract

The Doñana Biosphere Reserve is one of the most recognized and studied natural areas in the world, hence the importance of having an openly accessible species checklist. Previous lists are outdated, often have limited taxonomic scope, lack clearly defined inclusion and exclusion criteria, or are practically inaccessible. Here, an updated checklist of vertebrate species for the Doñana Biosphere Reserve based on explicit spatial, temporal, and biological criteria is presented, reviewed and complemented by expert zoologists. The resulting inventory includes 700 vertebrate species, with birds (60%) and fishes (26%) being the most diverse groups. Seven species are considered extinct in the region, two are possibly extinct (i.e., have not been recorded in the past decade), and 32 are classified as exotic. This updated and curated checklist fills a critical gap in regional biodiversity knowledge and establishes a robust foundation for future monitoring, research, and conservation efforts in this ecologically unique and increasingly threatened area of southwestern Europe.

## Introduction

The Doñana Biosphere Reserve (hereinafter DoñanaBR) is located in the southwest of the Iberian Peninsula, near the mouth of the Guadalquivir River, and comprises three main environments. First, an extensive dune system, with approximately 50,000 ha, formed by stabilized and mobile dunes, with a canopy of xerophytic and hygrophytic scrub with cork oaks and pine trees, dotted with thousands of temporary ponds (Rodríguez-Ramírez et al. 1996; Díaz-Paniagua et al. 2015), surrounded by agricultural areas. Secondly, the marismas (i.e., seasonally flooded freshwater marshes) of the Guadalquivir, which historically occupied an area of approximately 150,000 ha, but had their surface area reduced to one-fifth (30,000 ha). They are a flat expanse of clay, which floods with the autumn or winter rains and dries out with the drought and strong summer heat typical of the Mediterranean climate (Green et al. 2017; Román 2024). This environment is so peculiar that foreign visitors in the early 20^th^ century used the Spanish word ‘marismas’ to refer to this area because there was no equivalent word in English (Chapman 1884). The marine zone, together with the Guadalquivir estuary, is the third major environment of the DoñanaBR. It consists of a shallow continental shelf (<50 m), with sandy and silty bottoms, high biological productivity, and a sandy coast with a beach almost 60 km long (Ruiz and García-Lafuente 2006; Navarro and Ruiz 2006).

For centuries, the terrestrial environments were inhospitable to human presence and activity, as neither the sandy areas nor the marismas could be cultivated with traditional systems, resulting in the absence of significant settlements in the region. This wild character did not go unnoticed by the hunter-naturalist travelers of the 19^th^ century, who found in Doñana one of the last strongholds of untamed nature in Europe (Chapman and Buck 1910). The environmental value of the region further gained international recognition following the success of the book “Portrait of a Wilderness” (Mountfort 1958), which contributed to the declaration of the National Park in 1969.

During the 20^th^ century, two opposing processes shaped the current landscape of the Doñana region. First, the area was impacted by direct human pressures, particularly land-use changes, with the establishment of intensive berry crops in the sandy areas and other irrigated crops in the drained marshes, such as rice, as well as a great development of beach tourism and an increase of religious pilgrimage activities (Green et al. 2024). Second, in contrast, the region has received a large number of recognitions in Europe: National Park (1969), UNESCO Biosphere Reserve (1980), Ramsar Site (1982), Natural Park (1989), Special Protection Area for Birds-SPA (1988), World Heritage Site (1994), Fishing Reserve at the mouth of the Guadalquivir (2004), Mediterranean Biogeographic Site of Community Importance-SCI (2006), Special Area of Conservation-Natura 2000 (2012), Marine Protected Area (OSPAR) in 2014, and IUCN Green List certification (2015) among others. As a result of all these protection efforts, the protected area has expanded from 37,000 ha in 1969 to nearly 128,000 ha today. In 2013, the area of the DoñanaBR was expanded to include all protected areas and most of the municipalities of the region, integrating both the surrounding agricultural environments and rural urban centers.

A key factor that has kept the DoñanaBR in the international spotlight is its significance for the presence and conservation of vertebrates, particularly Palearctic waterbirds (de Felipe et al. 2024), as well as its role as a refuge for other endemic and emblematic fauna of the Iberian Peninsula, including species that were close to extinction, such as the Iberian lynx (*Lynx pardinus*) and the Iberian imperial eagle (*Aquila adalberti*) (Ferrer and Calderón 1990; Palomares et al. 1991). Despite this, the accessible knowledge on the vertebrates present in the area is limited, as many of the existing lists are outdated, do not explicitly define the criteria used for their elaboration, are published in grey literature, or cover only a limited subset of vertebrate groups. While these earlier works have contributed to understanding the area’s biodiversity, the absence of a comprehensive up-to-date inventory may limit research, conservation efforts, and effective management today. For all these reasons, the objective of this study is to update the checklist of vertebrate species recorded in the DoñanaBR.

### Taxonomic coverage

All taxa were identified at the species level and the taxonomy is defined according to the GBIF Backbone Taxonomy.

Kingdom: Animalia.

Phylum: Chordata.

Class: Aves, Reptilia, Mammalia, Petromyzonti, Amphibia, Actinopterygii, Elasmobranchii.

Order: Testudines, Squatiniformes, Squamata, Suliformes, Gadiformes, Anseriformes, Artiodactyla, Gasterosteiformes, Galliformes, Pteroclidiformes, Passeriformes, Pelecaniformes, Chiroptera, Lagomorpha, Tetraodontiformes, Gruiformes, Apodiformes, Carnivora, Torpediniformes, Acipenseriformes, Cetacea, Soricomorpha, Carcharhiniformes, Mugiliformes, Atheriniformes, Aulopiformes, Rhinopristiformes, Myliobatiformes, Cuculiformes, Batrachoidiformes, Syngnathiformes, Beloniformes, Scorpaeniformes, Perciformes, Phoenicopteriformes, Anura, Lamniformes, Ciconiiformes, Falconiformes, Accipitriformes, Rajiformes, Cyprinodontiformes, Siluriformes, Erinaceomorpha, Columbiformes, Charadriiformes, Cypriniformes, Bucerotiformes, Zeiformes, Podicipediformes, Psittaciformes, Caprimulgiformes, Anguilliformes, Rodentia, Petromyzontiformes, Procellariiformes, Otidiformes, Strigiformes, Coraciiformes, Piciformes, Pleuronectiformes, Clupeiformes, Caudata, Gaviiformes.

### Spatial coverage

**Figure 1.**
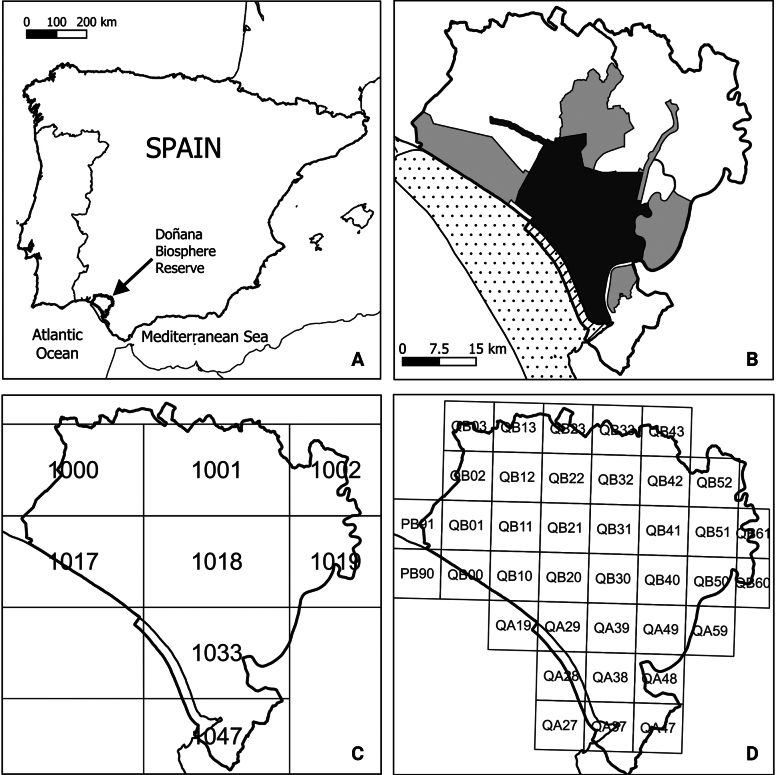
Study area. **A**. Location of the Doñana Biosphere Reserve (DoñanaBR) in the Iberian Peninsula; **B**. Boundaries of the DoñanaBR: the darker shade represents the core zone (mostly protected as National Park under Spanish legislation), the lighter grey corresponds to the buffer zone (mostly protected as Natural Park under Spanish legislation), the white areas within the Doñana area cover the transition zone, the striped polygon is the marine zone, and the dotted area represents the coastal strip; **C, D**. Grids used in distribution atlases to represent the distribution of the species: **C**. 1:50000 sheets of the Spanish Cartographic Service, and **D**. 10 × 10 km grids from which the species information has been taken.

The study area encompasses the DoñanaBR (269,000 ha; Fig. 1A). Nearly the entire area consists of terrestrial habitats. The core zone includes the National Park and the Rocina stream, while the buffer zone covers the rest of the protected area (Fig. 1B). The transition zone comprises the remaining territories of the municipalities that make up the Doñana BR. These municipalities are:

- Huelva province: Bonares, Rociana del Condado, Bollullos Par del Condado, Almonte and Hinojos; as well as the portions of Moguer and Lucena del Puerto that fall within protected areas.
- Sevilla province: Pilas, Villamanrique de la Condesa, Aznalcázar, Isla Mayor and La Puebla del Río.
- Cádiz province: Sanlúcar de Barrameda.

In contrast, only a small part of the DoñanaBR includes marine areas (Fig. 1B). These have been the least studied, and therefore, in order to provide a catalogue as complete as possible, we have included in this list the marine species recorded in a larger area, delimited by the Doñana coastal strip (Fig. 1B). The strip extends mainly off the coast of the DoñanaBR, up to the 50-m isobath, and has been defined according to homogeneous oceanographic characteristics, which differ from those of the rest of the Gulf of Cádiz (Ruiz et al. 2011).

### Temporal coverage

All vertebrate species recorded in the DoñanaBR from the beginning of the 20^th^ century to June 2025 are included in this checklist. The records draw on published literature (1960–2022), complemented by observation data and expert assessment up to mid-2025. The Doñana Biological Station also houses the Vertebrate Scientific Collection, a central repository for preserved specimens of many species recorded in the region (and beyond), some dating back to the late 1930s (Arrizabalaga 2024; Juste 2024a, b).

## Methods

**Figure 2.**
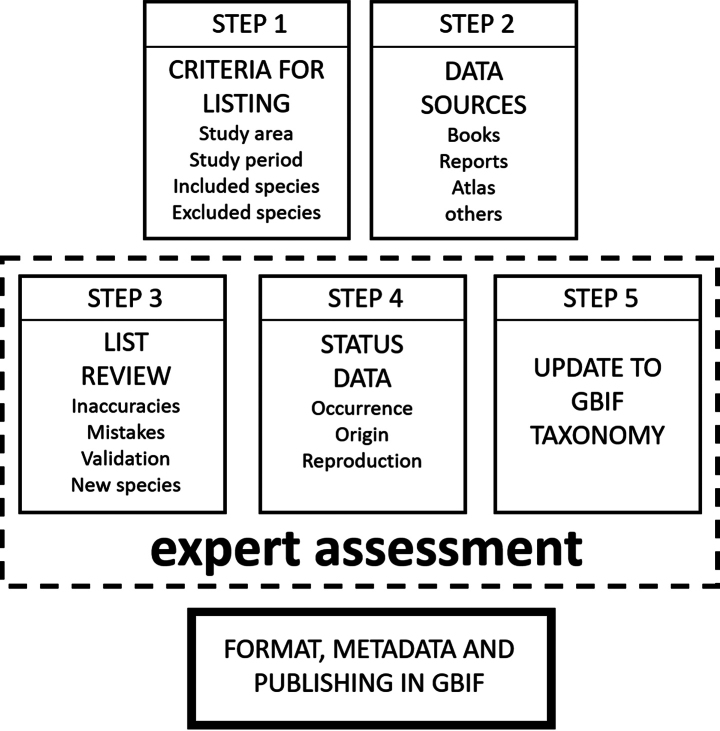
Process followed in the preparation of the checklist.

Five sequential steps were followed to compile this list (Fig. 2):

### Step 1. Defining the criteria for listing species

In addition to considering species with records that fall within the defined taxonomic, spatial, and temporal coverages, we applied a set of inclusion and exclusion criteria.

Criteria for species inclusion in the checklist:

- All sedentary, wintering, migratory, or accidental species are included, provided that their arrival has occurred naturally. Therefore, all exotic species that have reached the area naturally, from other established populations where they have been able to reproduce (such as birds arriving alongside other migratory species) are included.
- Native species extinct during the 20 ^th^ century in the DoñanaBR, as they may be observed again, either naturally recolonizing or through reintroductions in conservation projects.
- Exotic species introduced through releases or escapes within the study area, which have established breeding populations.

Criteria for species exclusion in the checklist:

- Exotic species that occur occasionally as a result of escapes or intentional releases in the region (such as wild animals kept as pets) and for which no established breeding populations are known.
- Exotic species that may have bred in the DoñanaBR but are no longer present.
- Domestic species, including those that breed in the area.

### Step 2. Identifying and compiling data sources

A key step in developing this checklist was the compilation of previously published inventories. These inventories were primarily published in books (Valverde 1960; Fernández 1974, 1982; García et al. 1989; Llandres and Urdiales 1990; Fernández-Delgado et al. 2000; García et al. 2000; Garrido et al. 2004; Díaz-Paniagua et al. 2005; García-Novo and Marín Cabrera 2005; García-Novo et al. 2007). The second source of information was texts including precise information on the general distribution of the species, such as atlases or field guides (Purroy 1997; Doadrio 2001; Palomo and Gisbert 2002; Pleguezuelos et al. 2002; Martí and del Moral 2003; Palomo et al. 2007; Salvador and Pleguezuelos 2013; Salvador et al. 2021; Molina et al. 2022). Finally, a few listings appear in reports, mostly unpublished (Ibáñez et al. 1995; Mateo et al. 1995; Román et al. 1999; Sobrino-Yraola et al. 2005; Chans et al. 2006) and, to a lesser extent, in scientific journal articles (Mountfort and Ferguson-Lees 1961; Collado et al. 1976; Gutiérrez-Expósito et al. 2012; Moreno-Valcárcel et al. 2020). These inventories may or may not include annotations, may cover only part of the DoñanaBR or only one of the vertebrate groups, or may be part of a distribution atlas; in the latter case, the grids considered in the different atlases are detailed in Fig. 1C, D.

### Step 3. Review of the list

The initial list was reviewed by expert zoologists familiar with the DoñanaBR fauna to validate the records and identify any inaccuracies, mistakes or misidentifications that may have been carried over from previous lists. In addition, new species not included in previous lists were added. Recent taxonomic changes have also been considered. These experts have been included as authors in this study.

### Step 4. Update of the status data

Expert knowledge was also used to update or provide new data on the status of occurrence, origin, and reproduction for species within the DoñanaBR, considering the following fields (the corresponding Darwin Core, dwc, term is given in parenthesis):

Means of establishment in the DoñanaBR (dwc:establishmentMeans): Refers to the autochthonous, introduced, or vagrant origin of the species in the DoñanaBR.

1. indigenous: The species is native or occurs naturally in the DoñanaBR; or, if the species was introduced, it was released in historical times (before 1500 CE).
2. exotic: The species is introduced in the DoñanaBR through either direct or indirect human activity after 1500 CE.
3. vagrant: The species is recorded once or sporadically, but it is known not to be native to the DoñanaBR.
4. uncertain: There is no certainty about the origin of the species in the DoñanaBR.

Reproduction status (dwc:occurrenceRemarks): Here, reproduction status refers to whether the species reproduces in the DoñanaBR.

1. Breeding: The species is known to breed or has bred within the boundaries of the DoñanaBR.
2. Non-breeding: The species does not reproduce within the boundaries of the DoñanaBR.
3. Uncertain: Species present during breeding season, yet reproduction has not been confirmed within DoñanaBR.

### Step 5. Update to GBIF Backbone Taxonomy

The taxonomy within the species list has been updated according to the GBIF Backbone (GBIF Secretariat 2023).

### Dataset description

The data are standardized and published via GBIF using three Darwin Core extensions and their associated terms:

- Taxon: taxonID, scientificName, kingdom, phylum, class, order, family, genus, specificEpithet, taxonRank, scientificNameAuthorship, taxonomicStatus, modified, language, license, rightsHolder, institutionCode, institutionID, datasetName, bibliographicCitation.
- Distribution: taxonID, locationID, locality, countryCode, eventDate, establishmentMeans, occurrenceRemarks, threatStatus.
- References: taxonID, bibliographicCitation, date, taxonRemarks.

Object name: Checklist of the vertebrate fauna of the Doñana Biosphere Reserve (Spain) – 2025.

Language: English.

Intellectual Rights: This work is licensed under a Creative Commons Attribution (CC-BY 4.0) License.

https://doi.org/10.15470/idnt5f.

## Results and discussion

The checklist compiles data on the presence of 700 vertebrate species recorded in the DoñanaBR since the beginning of the 20^th^ century, belonging to seven Classes, 64 Orders, and 192 Families. Five hundred forty-three species (77.6%) are considered indigenous, 32 species (4.6%) exotic, 115 (16.4%) vagrant, for ten species (1.4%) it was not possible to establish their status. Seven species are considered extinct in the region, six indigenous and one vagrant, and whose extinction has been global; two have not been recorded in the last ten years. In addition, 339 (48.4%) of the total number of species are known to reproduce in the DoñanaBR (Table 1).

**Table 1.** Number of species by taxonomic class, means of establishment (ORIGIN), and reproduction status (REPRODUCTION).

ORIGIN	Indigenous	Exotic	Vagrant	Uncertain
Petromyzonti	1	0	0	0
Elasmobranchii	24	0	0	0
Actinopterygii	146	11	0	0
Amphibia	11	0	0	0
Reptilia	23	2	3	1
Aves	296	17	95	9
Mammalia	42	2	17	0
REPRODUCTION	Breeding	Non breeding	Uncertain	Total species
Petromyzonti	0	1	0	1
Elasmobranchii	1	0	23	24
Actinopterygii	109	5	43	157
Amphibia	11	0	0	11
Reptilia	24	5	0	29
Aves	161	250	6	417
Mammalia	33	9	19	61

### Fish

Fish inhabit two main environments: freshwater fish are found in rivers, marshes, streams, channels, and ponds, and saltwater fish in the marine waters and in the Guadalquivir estuary. Freshwater and estuarine fish have been sampled and inventoried several times (Fernández-Delgado et al. 2000; Drake et al. 2002; Moreno-Valcárcel et al. 2020) unlike marine species which have been less studied as their environment remains the least prospected. In the list of saltwater fish we have included the species detected, or with probable presence, in the coastal strip up to the 50-meter isobath. These include eight species recorded in traditional fishing corrals (López-Amador and Ruiz-Gil 2010) and 37 recorded in the fish markets surrounding the DoñanaBR (Table 2). Two species of sharks, the basking shark *Cetorhinus maximus* and the blue shark *Prionace glauca*, have also been included, whose records come from stranded specimens on the beach.

**Table 2.** Fish species recorded in traditional fishing corrals and in fish markets.

Fishing corrals	Fish markets
*Aluterus monoceros*, *Diplodus cervinus*, *Muraena helena*, *Ranzania laevis*, *Scophthalmus maximus*, *Scorpaena porcus*, *Seriola dumerili*, and *Taeniurus grabatus*.	*Scyliorhinus canicula*, *Scyliorhinus stellaris*, *Sphyrna zygaena*, *Isurus oxyrinchus*, *Myliobatis aquila*, *Raja brachyura*, *Raja montagui*, *Raja undulata*, *Squatina squatina*, *Synodus saurus*, *Scomberesox saurus*, *Callionymus lyra*, *Naucrates ductor*, *Cepola macrophthalma*, *Coryphaena hippurus*, *Coris julis*, *Labrus bergylta*, *Labrus mixtus*, *Labrus viridis*, *Symphodus melops*, *Symphodus roissali*, *Symphodus tinca*, *Lobotes surinamensis*, *Chromis chromis*, *Cynoscion regalis*, *Sciaena umbra*, *Umbrina ronchus*, *Serranus scriba*, *Oblada melanura*, *Pagrus pagrus*, *Xiphias gladius*, *Monochirus hispidus*, *Dactylopterus volitans*, *Chelidonichthys lastoviza*, *Nerophis ophidion*, *Syngnathus typhle*, and *Zeus faber*.

Two freshwater species included in previous inventories were excluded from the current list, as the only known references are from nearby areas outside the limits of the DoñanaBR; these species are the rainbow trout *Onchorhynchus mykiss* and the northern pike *Esox lucius*.

Considering all these factors, the total list of fish species comprises 182 species, including one from the Class Petromyzonti, 24 Elasmobranchii, and 157 Actinopterygii. Two fish species are considered extinct in the DoñanaBR: the European sea sturgeon, *Acipenser sturio* (last record in 1992; Elvira and Almodovar 1993), and the three-spined stickleback, *Gasterosteus aculeatus* (last record in 1975; Hernando 1975). Eleven exotic species have been recorded, all of which are freshwater species. Finally, there is a great lack of knowledge about marine species, which is reflected in the high number of uncertainties about whether or not they breed in the DoñanaBR (Table 1).

### Amphibians

In the DoñanaBR there are 11 species of amphibians, three urodeles and eight anurans, all autochthonous, resident, and breeding in the region (Table 1). Early inventories also included the presence of the fire salamander, *Salamandra salamandra* (Fernández 1974, 1982; Collado et al. 1976); however, this species was excluded from the lists due to misidentification with other urodele species (Valverde 1960, 1967).

### Reptiles

The reptile fauna includes 29 species of which nine are turtles, five of them marine; eight are snakes, one of them a viper; and 12 are saurian, of which six are lizards, two are geckos, two are skinks, one is a worm lizard, and one is a chameleon. The presence of the Spanish sand racer *Psammodromus hispanicus* has recently been reported in nearby areas of the eastern part of the Guadalquivir (Doniol-Valcroze et al. 2023). This species has a morphology very similar to that of the west Iberian sand racer *Psammodromus occidentalis*, so genetic analysis would be necessary to confirm the presence of this former species in the DoñanaBR, potentially increasing the species count to 30.

Two species are considered exotic (Table 1). On the one hand, the Mediterranean chameleon, *Chamaeleo chamaeleon*, is present in the DoñanaBR as a result of releases made throughout the 20^th^ century and its population was probably reinforced with new introductions in urbanized areas associated with beach tourism (Díaz-Paniagua and Mateo 2015). On the other hand, the pond slider, *Trachemys scripta*, established a breeding population in the ponds near visitor centers within the park in the 2000’s, probably as a result of pet releases (Pérez-Santigosa et al. 2006). After a continuous trapping effort carried out during subsequent years, it can now be considered eradicated within the national park, although sporadically released individuals have occasionally been detected and removed. Nevertheless, given the ability of this species for successful breeding in these habitats, the possibility of its occurrence and the establishment of new populations in other areas of the DoñanaBR cannot be ignored, particularly in the proximity of urbanized areas. Previous inventories also included the Chinese softshell turtle, *Pelodiscus sinensis*, as some individuals were observed in rice paddy areas (Pleguezuelos et al. 2002); however, there is no certainty that this species has reproduced within the DoñanaBR, so it has been excluded from this checklist.

There is also a population of *Testudo graeca*, of uncertain origin, whose presence was first documented in 1765 (Andreu et al. 2000). Recent genetic analyses do not differentiate the DoñanaBR population from the autochthonous populations in south-eastern Spain, considering that further analyses are needed to clarify the origin of the Doñana tortoises and their relationship with south-eastern Iberian and North African populations (Graciá and Giménez 2015).

Records of the five species of sea turtles are mainly from beach strandings. Three of these species are considered vagrant, the green sea turtle *Chelonia mydas*, the hawksbill turtle, *Eretmochelys imbricata*, and the Kemp’s ridley sea turtle, *Lepidochelys kempii*, as there are very few records.

### Birds

Birds are the group with the highest number in previous inventories and, probably, the one with the highest number of errors. Among the species usually included in previous inventories but excluded from the current one are five occasional species whose records were made in nearby areas, but outside DoñanaBR, 13 species that have been considered misidentifications, and 28 exotic species that have escaped into the area but have not established breeding populations (Table 3).

**Table 3.** Excluded and newly recorded bird species in the current checklist. Excluded bird species were recorded in previous inventories but were removed for specific reasons (records made outside the DoñanaBR, misidentifications, or non-breeding exotics). These species are grouped according to the reason for exclusion. New bird species are those included for the first time.

Bird species excluded from the current checklist		
Outside DoñanaBR	Misidentifications	Escaped into the area and not breeding
*Asio capensis*, *Chersophilus duponti*, *Emberiza aureola*, *Lanius collurio*, and *Melanocorypha yeltoniensis*.	*Anthus petrosus*, *Aquila rapax*, *Bucephala islándica*, *Calandrella raytal*, *Ficedula semitorquata*, *Gavia arctica*, *Morus capensis*, *Oceanodroma castro*, *Tringa solitaria*, *Periparus ater*, *Phalacrocorax aristotelis*, *Sylvia nisoria*, and *Sylvia sarda*.	*Acridotheres tristis*, *Agapornis fischeri*, *Amadina fasciata*, *Amazona aestiva*, *Anas bahamensis*, *Anser cygnoides*, *Ara ararauna*, *Ara macao*, *Ara severus*, *Balearica regulorum*, *Chauna torquata*, *Cyanoliseus patagonus*, *Erythrura gouldiae*, *Falco cherrug*, *Lagonosticta senegala*, *Melopsittacus undulatus*, *Nandayus nenday*, *Numida meleagris*, *Phasianus colchicus*, *Phoenicopterus chilensis*, *Phoenicopterus ruber*, *Ploceus cucullatus*, *Psittacus erithacus*, *Quelea quelea*, *Streptopelia orientalis*, *Tadorna cana*, *Taeniopygia guttata*, and *Torgos tracheliotus*.
Bird species added to the checklist		
*Circus macrourus*, *Aythya affinis*, *Pluvialis dominica*, *Chroicocephalus philadelphia*, *Sterna forsteri*, *Thalasseus albididorsalis*, *Thalasseus elegans*, *Xema sabini*, *Calidris subruficollis*, *Calidris tenuirostris*, *Stercorarius longicaudus*, *Mycteria ibis*, *Streptopelia senegalensis*, *Lanius isabellinus*, *Anthus hodgsoni*, *Oenanthe deserti*, *Phylloscopus borealis*, *Calonectris borealis*, and *Puffinus yelkouan*		

In contrast, 19 non-breeding species recorded in recent years in the DoñanaBR have been incorporated (Table 3), many of them validated in rarity committees or on Citizen Science platforms (De Juana 1990, 1997, 2003, 2004, 2005, 2006; Garrido et al. 2004; Dies et al. 2008; Gil-Velasco et al. 2019, 2022; Pardo de Santayana et al. 2023).

In the updated checklist, birds are the largest group, with 417 species. Of these, 313 are considered to be regularly present, 95 vagrant and nine of uncertain origin (Table 1).

There are three bird species considered extinct in the area: the slender-billed curlew, *Numenius tenuirostris*, whose extinction has been global (Buchanan et al. 2025); the Andalusian buttonquail, *Turnix sylvaticus*, whose last known European populations were likely those present in the DoñanaBR until the 1980s–1990s (Gutiérrez-Expósito et al. 2011); and the rook, *Corvus frugilegus*, which wintered in the area in the early 20^th^ century (Chapman and Buck 1910) but has ceased to do so in recent decades, likely due to a northward shift in its wintering range (Román and Gutiérrez 2008).

Seventeen exotic species that arrived naturally have been recorded in the DoñanaBR (Table 1), six of which breed in the area, all passerine species originating from central or southern Africa.

### Mammals

There are 61 known mammal species in Doñana: 27 terrestrial mammals (3 ungulates, 10 carnivores, 1 hedgehog, 2 lagomorphs, 8 rodents, 3 shrews); 23 marine mammals, including four seals and 19 cetaceans; and 11 bats. There is limited knowledge about the marine mammal fauna, as most cetacean records come from strandings of animals on the beach, including four species that did not appear in previous inventories: the Sei whale, *Balaenoptera borealis*, the orca, *Orcinus orca*, Sowerby’s beaked whale, *Mesoplodon bidens*, and Cuvier’s beaked whale, *Ziphius cavirostris*.

Four species of bats have been excluded from this inventory because we cannot trace the original records: they are easily confused with other species, or the available citation is not accurate. These species are the greater mouse-eared bat *Myotis myotis*, the lesser noctule *Nyctalus leisleri*, the greater horseshoe bat *Rhinolophus ferrumequinum*, and Mehely’s horseshoe bat *Rhinolophus mehelyi*. Previous inventories also included two cryptic species, the common pipistrelle *Pipistrellus pipistrellus* and the soprano pipistrelle *Pipistrellus pygmaeus*, which were considered a single species until the late 20^th^ century (Barratt et al. 1997). However, as there are no known records of the common pipistrelle in the DoñanaBR, it has been excluded from the updated checklist. Conversely, a new bat species, the lesser horseshoe bat *Rhinolophus hipposideros*, has been detected breeding in the DoñanaBR in recent years, and hence is added to the checklist.

Two species are considered exotic. The fallow deer, *Dama dama*, was probably introduced into the area centuries ago but became extinct before a second establishment of populations from new releases made in the late 19^th^ or early 20^th^ century (Granados 1987; Morenés 2005; García-Novo et al. 2007). The brown rat, *Rattus norvegicus*, is a species native to northern Asia and evidence of its presence indicates that it colonized Europe from the 18^th^ century onwards (Domínguez-García 2021).

During 2011, the presence of raccoons, *Procyon lotor*, was detected within the DoñanaBR likely as a result of pet releases in the area (Fernández-Aguilar et al. 2012). However, no reproduction was detected, and no animals have been observed since then, so the species has not been included in this checklist.

Two species of mammals are considered extinct in the DoñanaBR: the wolf, *Canis lupus*, and the lesser mouse-eared bat, *Myotis blythii*. Until the 1960s, some wolves still appeared in the region, dispersing from the Sierra Morena populations (Valverde 1960). These populations have since disappeared (Clavero et al. 2023), making the arrival of wolves to the DoñanaBR improbable at present. In contrast, there are references to the presence of groups of lesser mouse-eared bats in the 1950s in the Doñana Palace, as well as occasional subsequent records (Ibáñez et al. 1995), although the species has not been observed in the area since. Finally, two other species have not been reliably recorded for more than ten years, namely the European wildcat, *Felis silvestris*, and the European polecat, *Mustela putorius*. Both were already rare species at the end of the 20^th^ and beginning of the 21^st^ century (Román et al. 1999; Soto-Navarro and Palomares 2014).

### Free-ranging domestic animals

The Doñana National Park, within the Biosphere Reserve, has traditionally been managed as a cattle-raising territory. There are several species of domestic animals under different degrees of management, mainly horses (*Equus caballus*), cows (*Bos taurus*), sheep (*Ovis aries*), goats (*Capra hircus*), chickens (*Gallus gallus* f. *domesticus*), dogs (*Canis lupus* subsp. *familiaris*), and cats (*Felis catus*), many of them belonging to local autochthonous breeds (Castroviejo 1993). Ungulates, especially cattle and horses, are managed in semi-freedom, with their movements limited on large farms by cattle fences (Botting et al. 2025). From the end of the 19^th^ century until the 1980s, a group of feral camels, *Camelus dromedarius*, bred in the area (Duque 1977). In recent years, feral dogs have also bred within the protected area (Gutiérrez-Zapata et al. 2024). Among the birds, the Muscovy duck *Cairina moschata* var. *domestica*, and the common pigeon *Columba livia* f. *domestica*, breed in the area. All these species have an important impact on the ecosystem, through vegetation control, disease transmission, or predation, among others (Soriguer et al. 2001; López et al. 2009; Gutiérrez-Zapata et al. 2024). However, they have not been included in this checklist because they are domestic species.

## Concluding remarks

Checklists are essential baseline tools for documenting the existing species richness in an area, monitoring the possible presence of exotic species, and recording information on the extinction or arrival of new species (Droege et al. 1998). They are living documents that evolve as knowledge expands. They are also subject to revisions and updates, which may result in the removal of species due to inaccuracies or misidentifications. These revisions can further be supported by voucher specimens stored in central public repositories, which provide physical material to revise and/or validate taxonomic identifications (Shaffer et al. 1998; Lister 2011). Ultimately, checklists underscore the importance of a territory for biodiversity conservation and provide stakeholders – managers, researchers, and the general public – with essential tools for understanding, valuing, and protecting these areas.

The Doñana Biosphere Reserve, despite being one of the most internationally recognized and best studied natural areas in the world, lacked an accessible list based on explicit criteria. This updated and comprehensive checklist of vertebrate species is a significant contribution to local and regional biodiversity knowledge, providing a solid baseline for future biodiversity monitoring. Its value lies not only in its comprehensiveness but also in the taxonomic verification and validation, achieved through the expertise of local researchers and managers.

We hope this study will inspire the development of additional checklists for the DoñanaBR, expanding to other taxonomic groups, and open new avenues for understanding the diversity, population dynamics, biogeography, and ecology of this unique and threatened area of southwestern Europe.

## Additional information

### Use of AI

No use of AI was reported.

### Author ORCIDs

Jacinto Román https://orcid.org/0000-0003-0675-9432

José L. Arroyo https://orcid.org/0000-0002-7605-5599

Giulia Bastianelli https://orcid.org/0000-0002-0139-3067

Javier Calzada https://orcid.org/0000-0001-5701-2238

Miguel Clavero https://orcid.org/0000-0002-5186-0153

Carmen Díaz-Paniagua https://orcid.org/0000-0002-6717-0173

Carlos Ibañez https://orcid.org/0000-0003-1181-7641

Javier Juste https://orcid.org/0000-0003-1383-8462

Jesús Nogueras https://orcid.org/0000-0002-5411-8173

Eloy Revilla https://orcid.org/0000-0001-5534-5581

Rubén Rodríguez https://orcid.org/0000-0002-1779-0075

Eduardo José Rodríguez-Rodríguez https://orcid.org/0000-0002-1170-0788

Guyonne F. E. Janss https://orcid.org/0000-0003-4318-6602

Zulima Tablado https://orcid.org/0000-0003-4520-9417

Marcello D’Amico https://orcid.org/0000-0002-0573-9184

Laetitia M. Navarro https://orcid.org/0000-0003-1099-5147

### Data availability

The checklist is available in GBIF (https://doi.org/10.15470/idnt5f).
